# Comparisons of functional movements and core muscle activity in women according to Pilates proficiency

**DOI:** 10.3389/fphys.2024.1435671

**Published:** 2024-11-25

**Authors:** Hyun Seo Ko, Hyung-Ung Jung, Tae-Young Park, Jong-Kook Song, Junsig Wang, Hyun Chul Jung

**Affiliations:** ^1^ Department of Physical Education, Graduate School, Kyung Hee University, Yongin, Republic of Korea; ^2^ Department of Sports Medicine, College of Physical Education, Kyung Hee University, Yongin, Republic of Korea; ^3^ Sports Science Research Center, College of Physical Education, Kyung Hee University, Yongin, Republic of Korea; ^4^ Department of Sports Coaching, College of Physical Education, Kyung Hee University, Yongin, Republic of Korea

**Keywords:** core muscle activity, electromyography, functional movement, muscle co-contraction, Pilates

## Abstract

**Introduction:**

This study aims to investigate the differences in functional movements and core muscle activities between experienced and novice practitioners during Pilates exercises.

**Methods:**

Thirty-eight participants were recruited for the study, comprising 19 experienced and 19 novice Pilates practitioners. Participants performed functional movement screening (FMS) tests and six Pilates exercises at the basic, intermediate, and advanced levels. Surface electromyography (EMG) was utilized to measure muscle activity at four sites: right rectus abdominis (RA), external oblique (EO), multifidus (MU), and longissimus (LO). Mean EMG activity, co-contraction indices, and duration of core muscle activation were analyzed using independent t-tests to examine the differences between groups. Cohen’s d was used to calculate effect sizes based on the standard deviations of the groups. Statistical significance was set at *p* < 0.05.

**Results:**

The experienced practitioners scored significantly higher in total FMS scores and in four sub-units of the FMS scores compared to the novice group (*p* ≤ 0.01). Mean EO EMG activity was also significantly greater in experienced practitioners during all Pilates exercises (*p* < 0.05). Additionally, the RA/EO co-contraction index was higher in experienced practitioners during the ‘double leg stretch’ exercise (*p* = 0.02).

**Conclusion:**

The results suggest that experienced Pilates practitioners have superior functional movement abilities and greater core muscle activation, particularly in the EO muscle group, compared to novice practitioners. These findings may assist Pilates instructors in refining instructional strategies to cater to different skill levels and enhance training effectiveness.

## 1 Introduction

Pilates has been widely used in recent years to improve trunk stability by strengthening core muscles (Kim and Kim, 2022). This exercise was developed by Joseph Pilates (1886–1967) during the First World War with the aim of rehabilitation from injuries (Petruk et al., 2021). Pilates follows the following six main principles: centering, concentration, control, breathing, flow, and precision, all of which could contribute to establishing a strong core, often referred to as ‘powerhouse’ (Petruk et al., 2021; Werba et al., 2017). Pilates emphasizes ‘control’ of our body to activate local muscles to support the lumbar spine and reduce pressure on the peripheral joints. By following these principles, Pilates exercise has been shown to improve functional movement and core stability (Šniurevičienė et al., 2022; Lima et al., 2021).

Functional movement is the ability to maintain and produce stability and mobility in daily activities (Lima et al., 2021). Multiple factors such as muscular strength, flexibility, endurance, coordination, balance, and movement efficiency are associated with performing functional movements (Beardsley and Contreras, 2014). It has been reported that improvement in functional movements via various exercise programs plays an important role not only in improving daily activity but also in preventing injuries (Clark et al., 2022). The functional movement screening (FMS) test is one of the well-known methods to assess movement patterns, including core stability and mobility of hip and shoulder joints (Beardsley and Contreras, 2014). Many studies have reported that functional movements are closely related to core stabilization (Zemková and Zapletalová, 2022). We believe that one of the core principles of Pilates, ‘centering,’ is linked to the improvement of functional movements. There are relevant studies examining the effects of Pilates on functional movements (Hornsby and Johnston, 2020). However, there remains a need for research examining the impact of Pilates on the functional movement of healthy adult females using quantitative measurement tools. This is crucial as it may provide valuable insights into the potential benefits of Pilates for promoting overall physical function and injury prevention in this population.

The ‘centering’ principle emphasizes the importance of activating core muscles, ensuring efficient muscle recruitment during movement (Marques et al., 2013). Activating the core muscles to sustain proper alignment enables them to function at an optimal length, which could lead to stabilization through the controlled movement of the spine and pelvis (Marques et al., 2013). Therefore, this could improve their capacity to utilize force effectively and minimize the onset of fatigue. Moreover, the ‘breathing’ principle supports the application of thoracic breathing, thereby easily utilizing and recruiting the core muscles through improved coordination (Kim and Lee, 2017). A recent study demonstrated transversus abdominis (TrA) activation in young and middle-aged women when performing five movements of Pilates (Tsartsapakis et al., 2023). However, despite the opinion that Pilates can strengthen the core muscles and firmly establish spinal stability, it is still unclear whether quantitative indicators such as EMG show the recruitment patterns of muscles stabilizing the spine.

Moreover, despite the growing popularity of Pilates among women, most existing studies have primarily focused on clinical populations such as individuals with lower back pain, which creates a significant gap in understanding its impact on healthy adult women engaged in preventative exercise. A recent systematic review and meta-analysis demonstrated the impact of Pilates exercises in reducing lower back pain and enhancing functional disability. The study showed that Pilates exercises were more effective compared to no exercise, with a large overall pooled effect size (SMD = −0.96, 95% CI: −1.51 to −0.41, *p* < 0.0001) and a moderate pooled effect size (SMD = −0.84, 95% CI: −1.27 to −0.42, *p* = 0.04) compared to non-specific exercises (Patti et al., 2024). Although these findings emphasize the therapeutic potential of Pilates on populations with lower back pain, they do not provide sufficient insights on the impact of Pilates on core muscle activity in healthy adult women population according to their proficiency.

We would like to address this gap in current studies as it could help develop evidence-based guidelines for Pilates instructors. The findings of this study may help instructors design effective programs for this population by providing insights into muscle activation patterns such as compensatory movements based on proficiency levels. Therefore, the purpose of this study was to examine differences in functional movements and core muscle activity during Pilates movements, according to proficiency in healthy young adult women. We hypothesized that there would be differences in functional movements, core and co-contraction muscle activities, and duration of activation during Pilates exercise between experienced and novice Pilates practitioners.

## 2 Methods

### 2.1 Participants

Participants were recruited through flyers distributed at the University. Based on our pilot data, the number of participants was determined by power calculation (Cohen’s d = 1.2, alpha = .05, power = .8). A total of 38 young adult women, aged 19–35 years old, voluntarily participated in the study and were classified as experienced (N = 19) and novice practitioners (N = 19) based on Pilates proficiency. Participants who had been participating in Pilates exercise for more than 2 years and practiced at least four times a week for a minimum of 60 min each session were classified as experienced practitioners. Those with less than 2 months of Pilates experience who practiced one or two times a week for 60 min each were classified as novice practitioners. The inclusion criteria aimed to minimize potential confounding variables that could impact functional movement and muscle activity outcomes (Menacho et al., 2010; Panhan et al., 2019; Krawczky et al., 2016). The inclusion criteria were (a) women with no engagement in other sports and exercise except Pilates exercise, (b) women with no history of disease or musculoskeletal injury, (c) absence of previous abdominal or orthopedic surgery of the lower back, (d) no medication, and (e) not pregnant. Participants who were unwilling to participate voluntarily were excluded. The study was conducted at the Growth and Aging Laboratory, which was fully equipped with the necessary measurement systems and provided space for performing Pilates movements. Prior to the experiment, we explained the study procedures and details about the risks and benefits of study participation. The study was approved by the University’s institutional review board (IRB).

### 2.2 Physique

Body height was measured using a stadiometer (T.K.K. Takei Scientific Ins Co., Japan). Participants stood upright with their heads straight, ensuring equal weight distribution on both feet, with their heels, buttocks, and the back of their heads touching the stadiometer. Height was measured while the participants looked straight ahead and recorded in increments of 0.1 cm. Body weight was measured using a digital scale (Cas 150A, Korea) while participants wore light clothing. Weight was recorded in increments of 0.1 kg. Body mass index (BMI) was calculated using the measured height and weight and recorded in kg·m^-2^.

### 2.3 Body composition

Participants’ body composition was measured by dual X-ray absorptiometry (DXA, QDR-4500W, Hologic, USA) to obtain the fat mass (FM), lean body mass (LBM), and body fat percentage. A previous study demonstrated intra-class correlation coefficients (ICCs) of DXA greater than 0.95 for body composition indicators (Jung and Song, 2018). Participants were positioned in a standardized manner following the Hologic scanner’s guidelines. The participants were positioned supine on the scanning table, the legs were internally rotated and secured with a band, and participants are instructed to maintain the fixed position during the scan. Additionally, participants were instructed to wear non-metallic sports bras or provided clothes that did not contain any metal components that could affect the results of body composition. The radiation exposure from a single DXA scan, which utilized the dual-energy X-ray absorptiometry technique, is significantly lower (approximately 10 μSv) than the annual natural radiation exposure (approximately 2–3 μSv). Fat mass, lean body mass, body fat percentage, bone mineral contents, and bone mineral density were reported in the study.

### 2.4 Functional movement screening (FMS) test

The functional movement screening (FMS) test is a widely used tool to assess and identify functional movement patterns. The FMS test has been validated in various studies (Beardsley and Contreras, 2014) and demonstrated high intra-rater reliability (0.88) and inter-rater reliability (0.90) (Sorenson, 2016). It consists of seven movement patterns, including deep squat, hurdle step, inline lunge, shoulder mobility, active straight leg raise, trunk stability push-up, and rotary stability movement pattern. Each pattern was evaluated three times by three experienced evaluators (>2 years more) who assessed it based on video recordings taken from the front and side angles. The FMS test and evaluation were carried out with the same evaluators throughout the study. The evaluation followed the basic instructions outlined in the FMS test protocol (Cook et al., 2006). A score of 3, 2, or 1 was assigned, with a score of 3 being the highest score, indicating the highest level of functional movement proficiency, a score of 2 related to having few dysfunctions, and a score of 1 reflecting many limitations. A score of 0 was assigned if pain appeared during the clearing test (Cook et al., 2006). By adhering to standardized guidelines and utilizing a multi-evaluator approach, we aimed to enhance the robustness of the FMS test results. A more detailed description of FMS™ can be found in Table 1.

**TABLE 1:** Functional movement screening (FMS) test.

Name	Instruction
Deep squat	Starting in a supine position, participants have the dowel placed behind their neck. They perform a squat movement with both hands holding a dowel, with feet positioned shoulder-width apart. This movement assesses flexibility, stability, and balance
Hurdle step	Starting in a standing, supine position, participants maintain a straight line from the waist to the leg while lifting one leg forward over the spring between the dowels. This movement evaluates stability and balance
Inline lunge	Starting in a standing position on the bar, participants hold a dowel in the hands on the back in a straight line. They place both feet in a straight line and bend both legs to perform a lunge movement. This assesses lower extremity strength, flexibility, and balance
Shoulder mobility	Starting in a standing position, participants extend their arms. Then, they elevate one arm and rotate it outward, and they rotate the other arm inward at the same time. This movement evaluates flexibility and the range of motion
Active straight leg raise	Starting in a lying position, participants lift one leg in a straight line. This movement assesses flexibility
Trunk stability push-up	Starting in a prone position, participants perform a push-up with hands placed on the floor and lift the whole body at once. This assesses upper body strength and stability
Rotary stability	Starting in a tabletop position, participants extend arms and legs in both sides at the same time, maintaining a straight line from the upper to the lower body. This movement assesses upper body strength and balance

### 2.5 Core muscle activity during Pilates movement

#### 2.5.1 Data recording

Surface electromyography (EMG) was utilized to measure the electrical activity of core muscles during Pilates movements. EMG data were recorded from four muscles: rectus abdominis (RA), external oblique (EO), longissimus (LO), and multifidus (MU) during Pilates movements. To collect EMG data from the core muscles, bipolar electrodes (Cometa Inc., MI, Italy; 2,000 Hz) with Ag/AgCl capture surfaces with diameters of 10 mm were positioned at the sites of the respective muscles. To ensure reliable localization, the electrodes were placed following the SENIAM (Surface ElectroMyoGraphy for the Non-Invasive Assessment of Muscles) guideline. The RA electrode was set 2 cm lateral and 2 cm above the umbilical scar. The EO electrode was placed above the anterior superior iliac spine at the level of the umbilical scar. The LO electrode was positioned 4 cm lateral to the level of L1, and the MU electrode was positioned 3 cm away from the line (midpoint) ranging from the spinous process of L1 to that of L5 vertebra (Rudolph and Snyder-Mackler, 2004).

For a normalization purpose, before the performance of the movements, we acquired EMG data for the core muscles (RA, LO, and MU) while the individuals performed a maximum voluntary isometric contraction (MVIC) on the isokinetic dynamometer (Cybex 770, HUMAC NORM, USA). Three trials of 5-s MVIC were conducted to normalize the EMG data, with a 10-s rest period between the MVIC trials to minimize fatigue. Participants were positioned in a supine position. Trunk flexor MVIC was acquired at 30°, and participants performed a forward flexion maneuver, while trunk extensor MVIC was measured at 60° by having participants maintain an extended position while leaning backward. For the external oblique MVIC, the participants performed oblique curl-up against manual resistance by the investigator (Ekstrom et al., 2007). Participants were given verbal encouragement during all MVIC trials to elicit the maximal effort.

#### 2.5.2 Analysis of the EMG signal

The raw EMG waveforms were band-pass filtered using a fourth-order Butterworth filter between 10 and 450 Hz to reduce contamination from movement artifacts. Additionally, to eliminate the effects of signal interference from nearby electronic sources, EMG waveforms were notch-filtered at 60 Hz using a fourth-order Butterworth filter. EMG waveforms were then full-wave rectified, the mean value was subtracted from the signal, and the resulting signal was subjected to a low-pass filter with a cutoff frequency of 10 Hz to obtain the EMG linear envelope. Individual muscle EMG amplitudes were calculated as the average linear envelope during Pilates exercises and then normalized to the peak amplitude for MVIC. Co-contraction indices were calculated using the equation below (Marshall and Murphy, 2003):

$$\begin{array}{ll} {CCI}_{m1:m2} & =ave\left\{\sum_{i-initial}^{i=final}\frac{\min\left\{{EMG}_{m1}\left(i\right),{EMG}_{m2}\;\left.\left(i\right)\right\}\right.}{\max\left\{{EMG}_{m1}\left(i\right),\right.{EMG}_{m2}\left.\left(i\right)\right\}}\right.\\ & \;\left({EMG}_{m1}\left(i\right)+{EMG}_{m2}\left(i\right)\right). \end{array}$$

In this equation, m1/m2 represent the two muscles, such as m1: RA and m2: EO, being analyzed; the initial/final were set to 1%–100% of the Pilates movements, from the starting point to the final point of the movement. In addition, min represents the EMG linear envelope values from the less active muscle group, and max represents the EMG linear envelope values of the more active muscle group at each time point. CCI was calculated during Pilates movements. CCIs were calculated for (m1:m2): RA:EO, MU:LO, RA:MU, and RA:LO.

In addition, the mean EMG signal of muscles exceeding 20% of MVIC was calculated during the Pilates movements, suggesting optimal core stability (Chan et al., 2017). All data analyses were conducted using the custom MATLAB code.

### 2.6 Pilates movements

The movements were chosen by the level of complexity and difficulty, and the level is based on the definition according to the classical Pilates method, which follows the principles and spirit of Joseph Pilates. The novices were required to have Pilates experience, and the experience was set to be less than 2 months, allowing a choice of exercises with greater difficulty. Participants performed a total of six Pilates movements including basic (Petruk et al., 2021), intermediate (Petruk et al., 2021), and advanced (Petruk et al., 2021) movements (Isacowitz, 2014) (Table 2). Participants were instructed on each movement and asked to practice three to five times for the purpose of familiarization. Then, the participants performed a single attempt of each movement with a 5-min rest interval between the Pilates movements to collect core muscle activity data.

**TABLE 2:** Pilates exercise.

Level	Name	Instruction
B	Chest lift	Supine, pelvis and spine neutral, knees flexed, and feet on the mat. Inhale to prepare. Exhale, contract abdominals, and flex thoracic spine. Inhale and maintain abdominal contraction. Exhale and roll upper body down to the mat
B	Spine stretch	Start from the sitting upright position, with arms forward. Inhale to prepare, and exhale when stretching the spine from the trunk flexion, starting with the head. Inhale and maintain the stretched position. Exhale and articulate the spine sequentially, from the tailbone to the head, returning to the starting position
I	Roll up	Start from a supine position keeping the lower limbs extended, with arms internally rotated a bit and flexed overhead. Inhale while the head goes up and the arms follow in line with ears. Exhale, make posteriorly tilted pelvis, and articulate the spine sequentially until the shoulder line goes with the pelvis. Inhale, remain, and exhale, and return to the starting position
I	Double leg stretch	Start from a supine position with a slight neck upward flexion and flexion of the hips and knees sustained by the hands on the knees, not entirely. Inhale and extend upper and lower limbs without touching the ground simultaneously. Exhale and return to the starting position
A	Teaser	Start in a tabletop position with arms straight and extended above the head and legs straight and behind on the ground. Upon initiation, inhale, and gather both arms and legs into a V-shape and contract both the erector spine and rectus abdominis muscles simultaneously, straightening the spine. Upon exhalation, tilt the pelvis posteriorly and sequentially articulate through the spine to return to the starting position
A	Jackknife	Start in a tabletop position with arms extended by the sides and legs straight and behind on the ground. Exhale and extend legs in a diagonal direction. Inhale and flex both legs to create a 90° angle with the ground. Upon exhalation, tilt the pelvis posteriorly and move the extended leg backward, over the head. Inhale, move the legs touching the ground with the plantar position of the foot, and extend the leg while straightening the spine and maintaining a 90° angle with the ground. At this point, the subject contracts both the trunk flexors and extensors, holds the position, and sequentially articulates the spine to return to the starting position

Descriptions of the Pilates movements. Abbreviations: B, basic; I, intermediate; A, advance.

### 2.7 Statistical analysis

The data were analyzed using SPSS for Windows version 26 (SPSS Inc., Chicago, IL, USA). All data were expressed as mean (M), standard deviation (SD), and 95% confidence interval (CI). Data normality was assessed by the Shapiro–Wilk test. After confirmation of normal distribution, an independent t-test was used to verify the difference in normalized muscle activations between the professionals and the novice groups. The effect size was calculated by Cohen’s d to describe the size of the effect in the FMS and EMG variables. Effect sizes were defined as small (d ≤ 0.4), medium (0.4 < d ≤ 0.75), and large (d > 0.75) (Cohen, 2013). The statistical significance level was set at .05.

## 3 Results

### 3.1 Anthropometric measurements

Anthropometric measurements of participants are shown in Table 3.

**TABLE 3:** Anthropometric measurements.

Variable	Experienced	Novice	*p-value*
Age (year)	29.2 ± 4.18	24.7 ± 2.40	<0.001
Duration (month)	33.9 ± 16.74	0.6 ± 0.84	<0.001
Body height (cm)	162.4 ± 5.77	161.4 ± 4.14	0.55
Body weight (kg)	53.9 ± 5.31	53.9 ± 7.23	0.97
Body mass index (kg·m^-2^)	20.4 ± 1.37	20.7 ± 2.39	0.67
Bone mineral density (g·cm^-2^)	1.1 ± 0.07	1.1 ± 0.05	0.73
Fat mass (kg)	13.9 ± 3.48	15.1 ± 3.86	0.37
Lean body mass (kg)	39.1 ± 3.60	38.1 ± 4.18	0.44
Body fat percentage (%)	26.2 ± 4.81	27.9 ± 4.97	0.26

Data are presented as mean ± standard deviation.

### 3.2 Functional movement screening test (FMS)

There were significant differences in total FMS scores between the groups, with experienced practitioners demonstrating significantly higher scores than novice practitioners (*p* = 0.001, ES = 2.88). Sub-unit analysis showed significantly higher scores and large effect sizes in the experienced group compared to the novice group (*p* ≤ 0.01) across four FMS categories: deep squat (ES = 1.99), hurdle step (ES = 2.77), inline lunge (ES = 4.78), and active straight leg raise (ES = 1.18). However, no significant differences in shoulder mobility, trunk stability push-up, and rotary stability were found between the groups. The results of the functional movement screening (FMS) test are reported in Table 4.

**TABLE 4:** Results of functional movement tests between groups.

Movement	Experienced (score)	Novice (score)	*p*-value	ES
Deep squat	2.9 ± 0.23	2.1 ± 0.52	<0.001	2.37
Hurdle step	2.9 ± 0.32	2.0 ± 0.33	<0.001	2.76
Inline lunge	3.0 ± 0.00	1.9 ± 0.23	<0.001	0.90
Shoulder mobility	2.9 ± 0.23	2.7 ± 0.45	0.08	0.62
Active straight leg raise	3.0 ± 0.00	2.4 ± 0.51	<0.001	2.28
Trunk stability push-up	2.0 ± 0.00	1.9 ± 0.23	0.32	0.46
Rotary stability	2.0 ± 0.00	1.8 ± 0.38	0.07	0.84
Total	18.7 ± 0.65	14.9 ± 1.75	<0.001	3.16

Data are presented as mean ± standard deviation. ES (effect size) values are interpreted as follows: small effect (0.2), medium effect (0.5), and large effect (0.8 or above).

### 3.3 Mean EMG activation level

The results of mean EMG activity are summarized in Table 5. There were significant differences in mean EMG activities during Pilates movements between the experienced and the novice practitioners. During all movements, the experienced showed significantly higher EO activity than the novice group (*p* = 0.005, *p* = 0.02, *p* = 0.005, *p* = 0.02, *p* = 0.006, and *p* = 0.03, respectively).

**TABLE 5:** Results of the mean EMG activation level (%) between the groups.

Movement	Muscle	Experienced	Novice	*p-value*	ES
Chest lift	RA	38.2 ± 14.41	40.4 ± 23.92	0.73	0.12
	EO	54.6 ± 18.27	40.26 ± 10.62	0.005	1.00
	MU	8.4 ± 12.22	13.8 ± 11.29	0.17	0.45
	LO	2.2 ± 4.99	20.91 ± 51.70	0.13	0.66
Spine stretch	RA	14.5 ± 11.89	17.3 ± 14.19	0.58	0.18
	EO	52.5 ± 18.83	39.8 ± 11.40	0.02	0.84
	MU	19.8 ± 14.43	22.2 ± 10.28	0.55	0.20
	LO	16.9 ± 9.60	38.2 ± 74.92	0.24	0.50
Roll-up	RA	38.9 ± 12.07	39.8 ± 21.63	0.89	0.05
	EO	54.4 ± 17.02	40.1 ± 12.15	0.005	0.98
	MU	23.4 ± 37.37	40.5 ± 62.35	0.31	0.34
	LO	17.1 ± 41.96	23.99 ± 52.06	0.65	0.15
Double leg stretch	RA	38.0 ± 11.62	41.4 ± 22.50	0.55	0.20
	EO	54.2 ± 18.09	41.0 ± 15.24	0.02	0.79
	MU	8.5 ± 13.86	13.9 ± 13.37	0.23	0.40
	LO	4.3 ± 8.24	16.6 ± 39.65	0.19	0.52
Teaser	RA	36.1 ± 7.73	39.3 ± 21.29	0.55	0.22
	EO	56.5 ± 13.91	42.2 ± 16.66	0.006	0.93
	MU	22.9 ± 39.20	20.6 ± 11.06	0.81	0.09
	LO	15.50 ± 20.22	28.6 ± 51.24	0.31	0.37
Jackknife	RA	37.2 ± 8.77	38.6 ± 23.95	0.81	0.08
	EO	53.3 ± 13.02	41.7 ± 17.59	0.03	0.76
	MU	27.7 ± 14.84	38.3 ± 22.28	0.09	0.57
	LO	23.6 ± 13.71	32.3 ± 34.64	0.32	0.36

Data are presented as mean ± standard deviation. Abbreviations: RA: rectus abdominis; EO: external oblique; MU: multifidus; LO: longissimus; ES: effect size. ES values are interpreted as follows: small effect (0.2), medium effect (0.5), and large effect (0.8 or above).

### 3.4 Co-contraction EMG activation level

Table 6 shows the results of co-contraction indices between experienced and novice practitioners. During ‘double leg stretch,’ the professionals demonstrated a significantly higher RA/EO co-contraction level compared to the novice (*p* = 0.02).

**TABLE 6:** Results of the co-contraction EMG activation level (%) between the groups.

Movement	Muscle	Experienced	Novice	*p-value*	ES
Chest lift	RA/EO	15.4 ± 8.63	13.1 ± 3.92	0.29	0.37
	MU/LO	14.6 ± 63.78	4.3 ± 5.88	0.32	0.46
	RA/MU	6.4 ± 5.94	7.1 ± 6.32	0.73	0.11
	RA/LO	4.6 ± 3.74	6.1 ± 5.53	0.32	0.33
Spine stretch	RA/EO	8.5 ± 7.90	19.5 ± 45.76	0.32	0.41
	MU/LO	7.4 ± 5.15	7.8 ± 7.18	0.84	0.07
	RA/MU	5.8 ± 9.13	8.1 ± 10.64	0.48	0.24
	RA/LO	3.9 ± 5.03	7.2 ± 7.53	0.14	0.50
Roll up	RA/EO	13.4 ± 5.87	15.4 ± 8.62	0.40	0.28
	MU/LO	7.8 ± 15.63	6.4 ± 6.42	0.70	0.14
	RA/MU	6.2 ± 4.69	8.9 ± 6.58	0.15	0.48
	RA/LO	3.9 ± 2.37	5.9 ± 5.66	0.15	0.52
Double leg stretch	RA/EO	17.5 ± 7.24	12.6 ± 4.55	0.02	0.83
	MU/LO	2.9 ± 4.47	4.3 ± 6.77	0.48	0.25
	RA/MU	4.4 ± 4.89	4.9 ± 6.11	0.74	0.11
	RA/LO	2.8 ± 2.43	5.3 ± 6.27	0.12	0.57
Teaser	RA/EO	15.6 ± 5.74	15.5 ± 9.96	0.98	0.01
	MU/LO	5.1 ± 5.31	8.0 ± 7.06	0.32	0.46
	RA/MU	4.9 ± 5.80	7.6 ± 7.25	0.22	0.41
	RA/LO	4.0 ± 2.84	6.7 ± 6.32	0.09	0.59
Jackknife	RA/EO	14.6 ± 0.07	11.7 ± 5.90	0.49	0.23
	MU/LO	7.8 ± 5.52	6.8 ± 6.04	0.48	0.24
	RA/MU	6.7 ± 5.49	9.2 ± 7.32	0.28	0.36
	RA/LO	4.3 ± 3.90	7.3 ± 6.80	0.16	0.50

Data are presented as mean ± standard deviation. Abbreviations: RA/EO, co-contraction of rectus abdominis and external oblique; MU/LO, co-contraction of multifidus and longissimus; RA/MU, co-contraction of rectus abdominis and multifidus; RA/MU, co-contraction of rectus abdominis and multifidus; RA/LO, co-contraction of rectus abdominis and longissimus; EO/MU, co-contraction of external oblique and multifidus; EO/LO, co-contraction of external oblique and longissimus. ES (effect size) values are interpreted as follows: small effect (0.2), medium effect (0.5), and large effect (0.8 or above).

### 3.5 Duration of EMG activation

Table 7 shows the results of the duration of EMG activation of the core muscles during the Pilates movements between experienced and novice practitioners. During ‘spine stretch’ and ‘jackknife,’ the novice group demonstrated a longer muscle activity duration of MU than the experienced group (*p* = 0.04). In addition, the novices presented a longer time of EMG activation than the experienced group while performing ‘roll up’ (*p* = 0.04).

**TABLE 7:** Results of EMG activation duration (sec) between the groups.

Movement	Muscle	Experienced	Novice	*p-value*	ES
Chest lift	RA	48.7 ± 23.88	48.5 ± 29.36	0.98	0.01
	EO	40.2 ± 30.63	46.1 ± 39.12	0.61	0.17
	MU	5.95 ± 13.98	11.1 ± 22.27	0.40	0.28
	LO	0.1 ± 0.32	10.1 ± 25.46	0.10	0.78
Spine stretch	RA	14.7 ± 28.23	24.2 ± 35.64	0.38	0.30
	EO	28.5 ± 34.62	40.4 ± 43.10	0.36	0.31
	MU	13.9 ± 21.19	25.5 ± 30.86	0.20	0.44
	LO	3.15 ± 4.96	19.6 ± 32.21	0.04	0.88
Roll-up	RA	36.4 ± 21.71	43.44 ± 30.41	0.42	0.27
	EO	37.8 ± 30.17	46.4 ± 39.71	0.46	0.25
	MU	5.1 ± 11.39	18.9 ± 25.15	0.04	0.76
	LO	0.3 ± 0.84	10.5 ± 24.11	0.07	0.82
Double leg stretch	RA	46.2 ± 21.63	50.2 ± 31.53	0.66	0.15
	EO	39.7 ± 31.62	46.1 ± 41.77	0.60	0.17
	MU	3.1 ± 8.58	10.6 ± 20.91	0.16	0.50
	LO	0.2 ± 0.59	10.5 ± 27.20	0.11	0.74
Teaser	RA	41.4 ± 23.64	47.4 ± 32.54	0.52	0.21
	EO	45.7 ± 29.50	51.2 ± 38.51	0.62	0.16
	MU	6.3 ± 18.50	16.5 ± 25.65	0.16	0.47
	LO	0.45 ± 0.76	11.09 ± 24.12	0.06	0.86
Jackknife	RA	41.1 ± 24.49	48.4 ± 31.82	0.43	0.26
	EO	43.5 ± 32.38	47.8 ± 42.74	0.73	0.11
	MU	8.01 ± 17.23	16.8 ± 23.13	0.19	0.43
	LO	2.0 ± 2.88	9.7 ± 15.44	0.04	0.84

Data are presented as mean ± standard deviation. Abbreviations: RA, rectus abdominis; EO, external oblique; MU, multifidus; LO, longissimus; ES, effect size. ES values are interpreted as follows: small effect (0.2), medium effect (0.5), and large effect (0.8 or above).

## 4 Discussion

This study aimed to examine functional movements and core muscle activities during Pilates exercises based on Pilates proficiency. The main finding of the present study indicates that the experienced practitioners, compared with the novice, demonstrated significantly higher FMS scores and co-contraction EMG activity during certain Pilates movements. Additionally, the novice group exhibited significantly longer duration of the core muscles EMG activity.

In the study, the FMS total score was significantly higher in the experienced practitioners than in the novice practitioners. A sub-analysis revealed that the experienced practitioners scored higher on ‘deep squat,’ ‘hurdle step,’ ‘inline lunge,’ ‘active straight leg-raise,’ and ‘shoulder mobility’ than novices. Previous studies found that Pilates exercise improves flexibility (Beardsley and Contreras, 2014; Ahearn et al., 2018; Pivotto et al., 2022), dynamic balance (Espinosa et al., 2018), and functional movement (Laws et al., 2017). These results align with the results of our study, particularly the significant differences between the groups in the ‘active straight leg-raise’ and ‘shoulder mobility’ scores. Furthermore, another study reported that recreational runners improved their FMS total score and other subtest scores after an 8-week Pilates intervention (Laws et al., 2017). The ability to perform these movements correctly indicates a well-developed sense of balance, mobility, and coordination, which are essential components of Pilates. These skills could be developed through consistent training of Pilates, which emphasizes concise control of movement, posture alignment, and centering and breathing techniques. However, we found no significant difference in “trunk stability push-up” and “rotary stability” between the groups. These movements require upper limb strength, balance, and stability, and achieving a 3 on these tests involves lifting the entire body simultaneously. It is possible that Pilates, which emphasizes movements centered around the deep muscles, does not contribute as effectively to the development of upper limb strength as traditional strength training. The nature of Pilates, with its focus on core stability and alignment, predominantly engages the deep muscles of the trunk, such as the transversus abdominis. This focus may limit the direct load placed on the upper limb muscles, leading to less upper body strength improvement in Pilates practitioners compared to those who engage in traditional upper body strength exercises. Given the emphasis on core engagement, Pilates exercises likely improve functional movement and core strength more than isolated upper limb strength, which could explain the lack of significant differences in the “trunk stability push-up” and “rotary stability” scores. Differences in participant populations across studies may also explain the varied outcomes. Although the previous study involved recreational runners, our study included Pilates instructors and novice practitioners. It is possible that the combination of running and Pilates in the previous study created complementary effects, enhancing overall benefits, rather than showing the isolated impact of Pilates.

Regarding core muscle activity, our results support our second hypothesis. A previous study found that the experienced practitioners exhibited higher core muscle activity than novices during Pilates exercises. The experienced group demonstrated mean EMG abdominal activity (transversus abdominis and internal oblique) that was approximately 50% or more of MVIC. This finding is consistent with ours as the experienced practitioners in our study showed a mean EMG activation level of EO over 50%, with significantly higher abdominal activity (54.6% MVIC) compared to novices (40.3% MVIC) during the chest lift movement. This suggests that experienced Pilates practitioners are better able to achieve and sustain core muscle contraction better than novices. Additionally, the experienced group displayed significantly higher EO activity than the novices across all six movements (*p* = 0.005, *p* = 0.02, *p* = 0.005, *p* = 0.02, *p* = 0.006, and *p* = 0.03, respectively). Similarly, a previous study reported greater RA and EO muscle activity during the roll-up exercise, emphasizing the role of Pilates breathing techniques (Barbosa et al., 2018; Silva et al., 2015). In Pilates, proper breathing is a fundamental principle, and thoracic breathing, which involves deep inhalation and controlled exhalation, plays a crucial role in the activation of the EO muscle (Kawabata and Shima, 2023). Thoracic breathing primarily engages the intercostal muscles, increasing EO activity, which is reflected in the EMG readings of experienced practitioners who have mastered the respiratory techniques used in Pilates (Andrade et al., 2022). A previous study noted that different breathing techniques in Pilates can lead to distinct muscle activation patterns, which may explain our findings (Barbosa et al., 2015). It is possible that proficiency in executing these movements, combined with proper breathing techniques, allows experienced practitioners to achieve more efficient and targeted muscle recruitment. In contrast, novices may struggle to maintain optimal muscle activation due to a lack of neuromuscular control and familiarity with the exercises, resulting in less efficient activation patterns. Conversely, a previous study found no significant differences in global abdominal muscle activity between experienced and novice practitioners when performing the ‘knee stretch’ in three different pelvic positions on a Pilates reformer (Lee, 2021). The use of different equipment may explain these discrepancies. The reformer apparatus provides assistance, making it easier for novices to perform movements and activate target muscles. In contrast, mat-based exercises require individuals to rely solely on their own effort, which may lead to variations in muscle activation based on the proficiency level.

Our study revealed a higher EO activation level compared to the RA activation level in the experienced group across all movements. This suggests distinct muscle activation patterns, with greater EO activation in experienced practitioners, likely due to their improved coordination of agonist and synergist muscles and breathing techniques (Lee et al., 2024; Park et al., 2024). Particularly, Pilates breathing techniques that expand the diameter of the thorax have been known to increase respiratory muscle engagement such as EO (Lee et al., 2024), resulting in improvement in respiratory function (Park et al., 2024). In addition, increased intra-abdominal pressure during Pilates movement can affect the spinal stability. The co-contraction of RA/EO muscles was also significantly higher in the experienced practitioners than in novices. Co-contraction between agonist and synergist muscles contributes to spine stability and movement accuracy. A previous study found similar results, reporting higher co-contraction EMG activity of the transversus abdominis and internal oblique in experienced practitioners than in novices (Espinosa et al., 2018). In our study, co-contraction of RA/EO EMG activity was particularly pronounced during the ‘double leg stretch’ movement, which requires coordinated engagement of these muscles to stabilize the spine and pelvis while extending and flexing the lower limbs. The complexity of the movement may explain the significant differences in RA/EO co-contraction between experienced and novice practitioners as the former are likely to have refined their motor and muscle control through practice. In addition, the ability to achieve high co-contraction levels may reflect enhanced trunk stability, which is critical for maintaining postural control and minimizing the risk of injury. Furthermore, the novice group exhibited significantly longer activation times for certain core muscles, such as the LO during the ‘spine stretch’ and ‘jackknife’ movements. These findings suggest that novices rely heavily on compensatory muscle activation due to insufficient recruitment of the primary movers, RA and EO. The long duration on activation of LO observed in novices during these movements indicates an over-reliance on this antagonist muscle, likely due to a lack of coordination and efficiency in engaging the primary core muscles. This finding aligns with a previous study on individuals with lower back pain, which reported similar compensatory muscle activation patterns (Silva et al., 2015). Our findings could suggest that novice practitioners may benefit from targeted training that focuses on proper core muscle engagement and minimizing the use of compensatory muscles, leading to improved effectiveness and reducing the risk of injuries.

In summary, the results of this study could contribute to the development of tailored Pilates programs based on the proficiency level. Understanding muscle recruitment patterns can help Pilates instructors provide more effective guidance. For instance, beginners may over-rely on lower back muscles during trunk flexion exercises, and instructors can use external cues to promote proper engagement of the core muscles. These findings highlight the potential to customize Pilates instruction to optimize muscle activation and minimize compensatory actions. Additionally, the use of objective EMG data provides a scientific basis for future Pilates exercise sequencing, relying on empirical evidence rather than subjective opinions.

The findings of the present study suggest as follows: (a) a comparison of muscle activation between groups with varying levels of Pilates proficiency using electromyography (EMG) as a quantitative measure, (b) an examination of the co-contraction activity between the primary muscle and the synergist and between the primary muscle and the antagonist, and (c) the use of two different exercises at each proficiency level to allow for diverse comparisons of muscle activation between experienced practitioners and novices. This approach provides a scientific basis for the future sequencing of Pilates exercises, relying on objective evidence rather than subjective opinions.

## 5 Limitations

There are several limitations in the study. First, this study recruited healthy adult women only, and the level of Pilates was determined based on frequency, time, and duration of Pilates. Second, age was not strictly controlled during recruitment as we prioritized participants based on Pilates experience. This should be considered in future studies for clarification of the findings. This limits the generalizability of the results. Third, the study was a cross-sectional study, and thereby, the study measured immediate outcomes. Thus, future studies should be considered for longitudinal effects. Fourth, we did not include the transverse abdominis, which could also contribute to core stability because superficial muscles cover its surface. An invasive EMG approach should be used to assess the transverse abdominis for a more precise comparison between the two groups. This should be considered in future study.

## 6 Conclusion

The findings of this study indicate that the experienced Pilates group showed greater functional movement abilities than the novice group. Furthermore, the experienced practitioners demonstrated significantly greater mean and co-contraction muscle activation, particularly in the EO muscle group. In addition, the novice group presented longer activation time of the posterior trunk muscles (MU and IL), which could suggest compensation patterns of muscle activation during Pilates movements. Our results may contribute to advancing our understanding of the neuromuscular mechanisms of Pilates exercise and the potential benefits it offers for functional movement, spine stability, and core muscle strength. Moreover, these insights from the findings can inform Pilates instructors in constituting more specifically tailored programs based on practitioner experience levels, thereby enhancing effectiveness of the exercise while ensuring safety. Further research is needed with more strict age controls to examine and validate observed differences in various populations and settings. In addition, longitudinal studies are essential to establish generalization and reach consensus on the benefits of the Pilates exercise.

## Data Availability

The raw data supporting the conclusions of this article will be made available by the authors, without undue reservation.
